# Impact of marine heatwaves for sea turtle nest temperatures

**DOI:** 10.1098/rsbl.2021.0038

**Published:** 2021-05-12

**Authors:** Graeme C. Hays, William J. Chivers, Jacques-Olivier Laloë, Charles Sheppard, Nicole Esteban

**Affiliations:** ^1^ Deakin University, Geelong, VIC 3216, Australia; ^2^ School of Electrical Engineering and Computing, University of Newcastle, Callaghan, NSW 2308, Australia; ^3^ School of Life Sciences, University of Warwick, Coventry CV4 7AL, UK; ^4^ School of Ocean Sciences, Bangor University, Menai Bridge LL59 5AB, UK; ^5^ Faculty of Science and Engineering, Swansea University, Swansea SA2 8PP, UK

**Keywords:** Chagos Archipelago, Granger causality testing, climate change, temperature-dependent sex determination, Hadley SST

## Abstract

There are major concerns about the ecological impact of extreme weather events. In the oceans, marine heatwaves (MHWs) are an increasing threat causing, for example, recent devastation to coral reefs around the world. We show that these impacts extend to adjacent terrestrial systems and could negatively affect the breeding of endangered species. We demonstrate that during an MHW that resulted in major coral bleaching and mortality in a large, remote marine protected area, anomalously warm temperatures also occurred on sea turtle nesting beaches. Granger causality testing showed that variations in sea surface temperature strongly influenced sand temperatures on beaches. We estimate that the warm conditions on both coral reefs and sandy beaches during the MHW were unprecedented in the last 70 years. Model predictions suggest that the most extreme female-biased hatchling sex ratio and the lowest hatchling survival in nests in the last 70 years both occurred during the heatwave. Our work shows that predicted increases in the frequency and intensity of MHWs will likely have growing impacts on sea turtle nesting beaches as well as other terrestrial coastal environments.

## 1. Introduction

Extreme weather events have massive ecological impacts across terrestrial, aquatic and marine habitats and can fundamentally shape ecosystems [1]. In the oceans, there is intense interest surrounding the ecological and socio-economic impacts of long-term ocean warming including discrete periods of prolonged anomalously warm water at particular locations, known as marine heatwaves (MHWs) [2,3]. MHWs can have a wide-range of impacts including major coral bleaching and mortality, seagrass and kelp die-offs, disease outbreaks and fisheries disruptions [4–7]. Impacts have been reported across the globe [8] and importantly even remote, relatively pristine areas that are far from localized anthropogenic impacts are not immune to the impacts of MHWs [9]. While the impacts of MHWs have been well documented for a range of coastal species and ecosystems, it is less well known if MHW impacts extend to adjacent terrestrial systems. For example, sea turtles nest on sandy beaches, i.e. close to the sea, and it is unknown whether their incubation conditions are impacted by MHWs. Indeed, there has been a call for a better understanding of how climate change will impact the biota of sandy beaches [10]. This question of MHW impacts on beaches is of conservation importance since incubation temperatures for sea turtles impact both the sex ratio of hatchlings as well as embryonic survival [11], giving rise to major concerns that generally warming conditions might cause the production of single-sex cohorts and so ultimately cause population extinctions [12].

In the austral summer of 2015/2016, a major coral-bleaching event associated with an MHW occurred in the Chagos Archipelago, a remote island group in the equatorial Indian Ocean that was previously known to host some of the most pristine coral reefs in the world [9]. Here, we take advantage of the recording of beach and water temperatures before, during and after this coral-bleaching event to consider the implications of the MHW for sea turtle incubation conditions and hence hatchling survival and sex ratios. We then use long-term temperature records to place this MHW in a multi-decadal context and consider the likely impacts of the increasing occurrence of MHWs for sea turtle nests, as well as other wildlife close to the sea.

## 2. Material and methods

Temperature loggers (Tinytag Plus 2 model TGP-4017, Gemini Data Loggers, UK, accurate to less than 0.5°C) were buried at nest depths (30, 50, 70 and 80 cm) to record the sand temperature every 4 h on a key nesting beach for hawksbill and green turtles on the southern coast of the island of Diego Garcia (7.42° S, 72.45° E) within the Chagos Archipelago (Indian Ocean). Diego Garcia hosts the highest nesting density of hawksbills and green turtles in the region. It is also an important nesting location for both turtle species in the context of overall nesting numbers across the western Indian Ocean [13]. Loggers were deployed to capture the extent of thermal variation across nesting zones on the beach, see [14] for details, and covered nesting depths for both hawksbill and green turtles. Loggers were placed at nest depths, but not inside nests. Across the range of depths, depth-related differences in sand temperature at this site are minimal, averaging 0.1 °C [14]. In total, we analysed data from 52 sand temperature loggers deployed between October 2012 and August 2019. Loggers recorded data for an average 19.9 months (s.d. = 6.6 months, min = 2.4 months, max = 35.6 months). Typically, there were between four and 10 loggers used in each mean monthly sand temperature calculation (median = 8). At this site, hawksbill turtles show a distinct nesting peak during October–February, and green turtles nest year-round with elevated activity during June–October [13].

Air temperature data for a 10 × 10 degree area around the Chagos Archipelago (2–12 °S and 66–76 °E) were obtained from the International Comprehensive Ocean-Atmosphere Data Set (ICOADS) through the National Center for Atmospheric Research (NCAR) (https://rda.ucar.edu/datasets/ds548.0/). The Enhanced ICOADS Monthly Summary Release 2.5 at 2-degree spatial resolution was used. Visual inspection showed that air temperatures were broadly homogeneous and so the exact area used in this analysis did not impact our overall conclusions. In addition, we extracted Hadley sea surface temperature (SST) data for the same geographic area from the UK Meteorological Office (http://www.metoffice.gov.uk/hadobs/hadisst/data/download.html). We used these freely available datasets, rather than local measurements, as they provide global coverage and have data extending back many decades. Hence these datasets can be used to reconstruct past conditions and our approach detailed here can be easily applied to nesting sites around the world.

Water temperatures on the coral reef at Diego Garcia were measured with Hobo U22 data loggers recording at 2 h intervals and accurate to less than 0.2°C. Loggers were secured at 15 m on the reef and protected against fish bites by short lengths of pipe. Rainfall data collected at Diego Garcia Airport were obtained from the airport meteorological station.

We calculated monthly means from our measurements of sand temperatures on beaches and reef water temperatures. This served to make these local measurements directly comparable with the ICOADS and Hadley datasets which are both supplied as monthly means. We investigated the relationship between our empirical sand temperatures and historical environmental variables using a stepwise multiple regression in which ICOADS air temperatures, ICOADS SST, Hadley SST, local reef water temperatures and precipitation were entered as predictor variables. Degrees of freedom in this analysis were adjusted for serial autocorrelation using the modified Chelton method [15]. We further explored the potential causal pathways present in our time series using a Granger causality test [16]. This approach represents a measure of forecasting over and above that provided simply by temporal correlations and helps point towards causal links.

For our predictive models of primary hatchling sex ratios and in-nest hatchling survival, (i.e. hatchling success), we assumed that metabolic heating within clutches averaged 1.1°C by the middle third of development (i.e. the period when sex is determined during incubation), as reported for hawksbill and green turtles in a recent review [17]. This value for metabolic heating was added to the mean monthly sand temperatures recorded at nest depths. We used the general relationships between incubation temperature and hatchling sex ratios and hatchling success [11], which assumed a pivotal temperature (i.e. the temperature at which the primary sex ratio is 50 : 50) of 29.1°C. In these relationships, greater than 99% males are produced at temperatures less than 26°C, greater than 99% females above 32°C and hatchling success declines to zero above 36°C. The same relationships were assumed for both species.

## 3. Results

**Figure 1.**
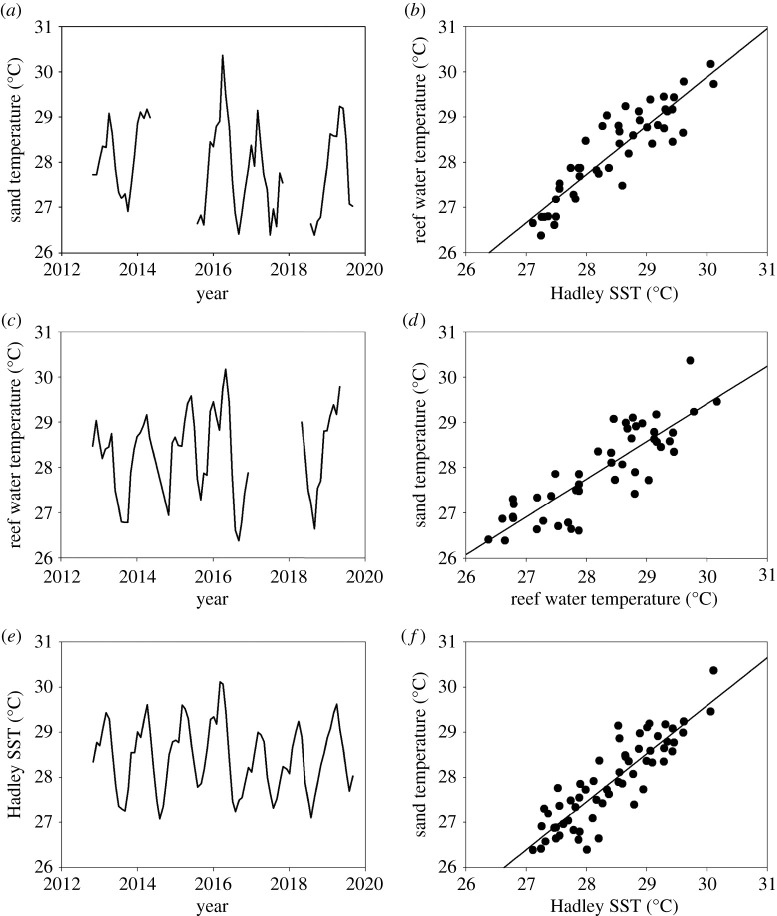
Time series of mean monthly (*a*) sand temperature at nest depths on Diego Garcia, (*b*) coral reef water temperature at 15 m on Diego Garcia and (*c*) Hadley SST measured more broadly across the Indian Ocean. The warmest months in all time series occurred in early 2016, corresponding with an MHW and major coral-bleaching event. The relationships between mean monthly (*d*) water temperature at 15 m on Diego Garcia and Hadley SST, (*e*) sand temperature at nest depths on Diego Garcia and water temperature at 15 m on Diego Garcia and (*f*) sand temperature at nest depths on Diego Garcia and Hadley SST. In each case, these regression equations were highly significant (*p* < 0.01) with *r*^2^ values of 0.81, 0.72 and 0.77, respectively. Due to logistical constraints of working at this remote nesting area, there was not a continuous rolling deployment of loggers, so some gaps when no loggers were deployed remain in our time series.

We obtained the mean sand temperature at nest depths for 61 separate months between October 2012 and August 2019. Across these 61 months, temperatures recorded (i) at nest depths on the nesting beach, (ii) at 15 m on local coral reefs and (iii) at the sea surface more broadly across the Indian Ocean (ICOADS SST and Hadley SST) all showed similar patterns and were all tightly correlated (figure 1), i.e. warm air and sea conditions in the ocean across that broad region were also reflected in warm water conditions on the local reef and in the sand on nesting beaches. Therefore, historic air and sea temperatures across this broad region can be used to reconstruct past sand temperatures. In all these temperature time series, the maximum temperatures were recorded in early 2016.

A stepwise multiple regression showed that Hadley SST alone was the best predictor for sand temperature: mean monthly sand temperature = 1.066 × Hadley SST – 2.375 (*F*_1,13.3_ = 193.1, *p* < 0.001, *r^2^* = 0.76). Put simply, a 1°C rise in SST translated into a 1.07°C rise in sand temperature (95% confidence interval = 0.91–1.22), so an MHW would translate into warmer turtle nest conditions. In addition, we found significant Granger causality (*p* < 0.05) in the relationship between mean monthly Hadley SST and mean monthly sand temperatures, i.e. when past values of SST were used in a regression model to predict future values of sand temperature (with a lag of 1 month) after adjusting for past values of sand temperature. This result further reinforces the view that variations in SST strongly influenced sand temperatures.

**Figure 2.**
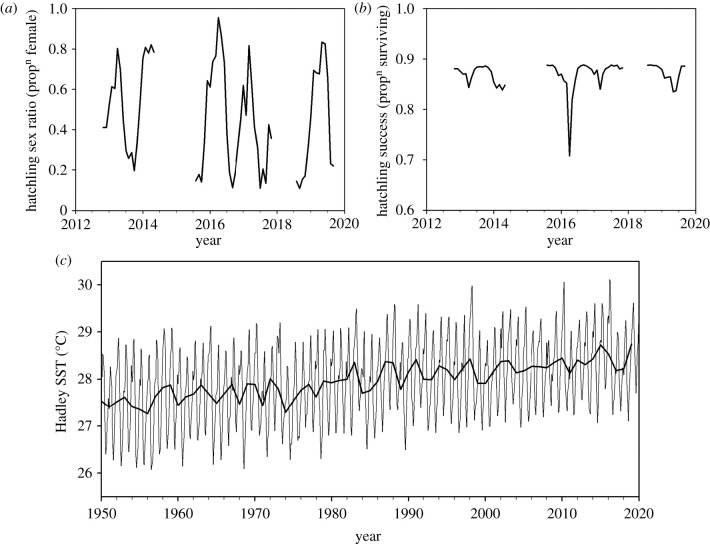
Based on the mean monthly sand temperature (figure 1*a*), the predicted (*a*) hatchling sex ratio and (*b*) hatchling success on Diego Garcia. In both cases, extreme values were predicted for March 2016, which was the warmest month in the time series. (*c*) The time series of mean monthly Hadley SST around the Chagos Archipelago. The fitted line is the mean annual temperature which increased linearly over time at a mean rate of 0.15°C per decade (*F*_1,68_ = 162, *p* < 0.001).

Modelled hatchling sex ratios and hatchling success predicted from the mean monthly sand temperature both showed impacts of the hottest temperatures in early 2016 (figure 2*a,b*). The modelled hatchling sex ratio varied seasonally from around 10–20% female in the coolest months (July and August) to around 80–90% female in the warmest months (February–March). The most extreme modelled female-biased hatchling sex ratio (95.6%) was predicted for March 2016. The modelled hatchling success was generally around 85–90%, but the lowest value (71%) was predicted for March 2016. The long-term (1950–2019) Hadley SST data show a 70-year trend of generally rising water temperatures in the region superimposed on the annual cycle, with the mean annual rise in temperature being 0.015°C (i.e. 0.15°C per decade) (figure 2*c*). March 2016 was the warmest month in this 70-year time series. There was a tendency for more extreme warmer temperatures as the time series progressed.

## 4. Discussion

We showed that an MHW that caused a major coral-bleaching event [9] also affected sand temperatures at sea turtle nest depths, with likely consequences for hatchling survival and sex ratios. This finding is noteworthy given the trend for increasing frequency and intensity of MHWs globally [8,18] and suggests that wider consideration should be given globally to MHW impacts on sea turtle incubation conditions. More broadly, our results suggest that MHW impacts are not confined to marine habitats, which have been the focus of previous studies [7].

The ecological impacts of the MHW for corals and sea turtle hatchlings were very different. During the 2016 coral-bleaching event in the Chagos Archipelago, coral cover on reefs dropped from around 40–50% to 10% on average and the reefs have been slow to recover [9]. Measured growth rates for several coral species in 2018–2019, following this bleaching event, were also comparatively low, suggesting prolonged effects of heat stress on coral physiology [9]. Concerns for the future of coral reefs in the region are heightened by the predicted increases in the frequency of severe bleaching events in the coming decades [19]. Set against the dire implications of MHWs for coral reefs in the region, our results suggest that for sea turtles the implications are likely to be far less severe, e.g. short-term changes in hatchling sex ratios and decreases in hatchling success.

The different impacts of the 2016 MHW on corals and turtle nests do not reflect differences between the respective temperature rises for coral reef and sandy beach environments. Rather the different responses reflect how close corals versus sea turtle nests were to their thermal tolerances. The reef water temperatures in the Chagos Archipelago are seasonally 28–29°C, which is very close to the thermal limits for corals and hence a slight increase in temperature can have very marked impacts [9]. By contrast, the general sand temperatures at nest depths on Diego Garcia are relatively low [14] and well within the thermal tolerances for developing embryos [20]. Nevertheless, our conclusion that MHWs can impact sea turtle nest temperatures has important broader implications. While sand temperatures at nest depths on Diego Garcia are relatively cool, in other locations much warmer sand temperatures often predominate and are of concern [12]. For example, across the globe, highly female-skewed sea turtle hatchling sex ratios dominate because incubation temperatures are generally well above the nominal pivotal temperature of 29°C [21]. In cases where incubation temperatures are already very high, the additional impact of MHWs is likely to be catastrophic, driving high hatchling mortality and reducing male production. Furthermore, if the trend for rising SSTs around the Chagos Archipelago and elsewhere continues as predicted [7], then the impact of future MHWs for hatchling sex ratios and hatchling mortality will grow. In short, we suggest that MHWs need to be considered an important and growing threat for sea turtles [22].

Given the concerns that we highlight of MHWs and rising temperatures for sea turtle incubation conditions, potential ways in which nest temperature rises might be mitigated need consideration, such as phenological shifts in nesting seasons [23] and artificially cooling nests [24]. Furthermore, our model could be improved if local measurements of metabolic heating were available. Direct measurements of hatchling sex ratios will also help refine estimates for the impact of MHWs.

The Hadley SST data suggest that the water temperatures associated with the 2016 coral-bleaching event were exceptional in the last 70 years, but also form part of a trend of warming conditions, which reiterates concerns for the future of coral reefs in the region [9,25]. It is also noteworthy that a previous coral-bleaching event was recorded in the Chagos Archipelago during the austral summer of 1997/1998 [9], as well as more broadly across the Western Indian Ocean [7], and the Hadley SST data again showed very warm conditions in March and April of that summer. Our findings also suggest, therefore, that around the world historical conditions in focal coastal areas might be reliably reconstructed using freely available broad-scale environmental measurements, albeit it is important, as done here, to first establish that broad-scale measurements reflect local conditions. In this way, observations in coastal areas might be placed into a much longer context of change occurring over many decades.

## Data accessibility

The data are provided in the electronic supplementary material [26].
